# Oral Health-related Quality of Life Among Denture Stomatitis Patients with Implant Overdenture Treated with Photodynamic Therapy

**DOI:** 10.3290/j.ohpd.b4997023

**Published:** 2024-02-20

**Authors:** Ghadeer I. Basunbul

**Affiliations:** Associate Professor and Consultant, Oral and Maxillofacial Prosthodontics Department, Faculty of Dentistry, King Abdul Aziz University, Jeddah, Saudi Arabia.

**Keywords:** dental implant, patient-centered outcome, patient satisfaction, photo-disinfection, quality of life, removable denture

## Abstract

**Purpose:**

This study aimed to assess the impact of photodynamic therapy (PDT) on the oral health-related quality of life (OHRQoL) among denture stomatitis patients with implant overdenture prostheses (IODs).

**Materials and Methods:**

The patients were recruited from a specialist dental practice according to selection criteria. The *Candida* spp. were identified and confirmed by the microbiological culture technique. *Candida* counts were estimated as colony-forming units (CFU/ml) at baseline, 15, 30, and 60 days. PDT was carried out twice a week with 72 h intervals for a period of 4 weeks. A structured questionnaire was used for data collection. It included the demographic details of the patients, including age, gender, education, marital and socioeconomic status (SES), oral habits, and smoking status. In addition, the Oral Health Impact Profile-EDENT (OHIP-EDENT) scale was added to assess the OHRQoL of all patients before and after PDT treatment. The data were analysed using descriptive statistics, the t-test and the Shapiro-Wilk test; statistical signifcance was set at p < 0.05.

**Results:**

At baseline, the overall mean *Candida* CFU/ml were quite high in the implant overdenture (IODs) samples, 37.12 ± 15.8, as compared to palatal mucosa samples with 5.1 ± 2.3. After PDT treatment, a statistically significant reduction was noted in the mean *Candida* CFU/ml on both surfaces at all follow-up visits. It was observed that all domains of OHIP-EDENT except for physical disability and handicap showed statistically significant improvement in mean scores after PDT treatment. FL, P1, P2, D2, and D3 had statistically significant mean score improvements of 2.2, 3.1, 2.2, 1.4, and 0.7, respectively. Furthermore, after PDT treatment, the total OHIP-EDENT score showed a statistically significant improvement of 11.6.

**Conclusion:**

PDT treatment has a positive impact on the OHRQoL for patients with denture stomatitis. It can be used as an effective treatment option for the treatment of denture stomatitis in IOD patients.

Denture stomatitis (DS) also recognised as denture sore mouth is a benign clinical condition often observed in denture-wearing patients. It may be described as chronic inflammation, with erythema of those oral mucosal tissues that are in intimate contact with the denture base.^18,30^ Its prevalence varies considerably across different countries and ranges from 15–77.5%, with an increased risk in females and the elderly.^18,31^ Denture stomatitis is more commonly observed beneath a maxillary removable denture, as the palatal mucosa is guarded against the washing effects of saliva and cleansing actions of the tongue.^29^

Denture stomatitis has multifactorial aetiology; nonetheless, in 90% of the cases, *Candida* species are implicated as the causative agent.^17,27,35^ Gauch et al^17^ identified five different *Candida* species in patients with DS, with *C. albicans* being the most prevalent one (78%). These are part of the commensal human oral microbiota.^17,27^ Xerostomia or altered salivary protein and inorganic composition, have been implicated to alter the oral microbiome composition that supports fungal overgrowth. A major role is also played by the compromised host-immune response or dietary deficiency.^7^ Other local predisposing factors that increase the risk of candida-associated DS include long-term tissue trauma due to poor fit or occlusal prematurity, denture defects, poor oral hygiene practices, and nocturnal denture use.^7,19,33^

Although the DS is usually painless, the inflammation can affect the oral health-related quality of life (OHRQoL) of the individuals, as the clinical signs include erythema and edema of the mucosal tissues, in combination with other subjective symptoms such as burning sensation, bad taste, halitosis and dry mouth.^24,27,28,33^ Moreover, angular cheilitis is often correlated with the presence of DS.^27^ It is presented with classic features of red, edematous, often painful patches of skin at the angles of the mouth. Furthermore, DS can be spread to other sites and have severe systemic consequences thus the need for early diagnosis and correct treatment is essential.^5,9,24^ Various local and systemic antifungal therapies had been used in the past for the treatment of candida-associated DS,^4,20^ but due to increased resistance, newer therapies including, photodynamic therapy and the use of nanoparticles have been suggested.^8,15,16^

Photodynamic therapy, involves a photosensitiser, a light source, and molecular oxygen. The photosensitiser acts by absorbing energy under the influence of light, altering the state of energy, and reacting with oxygen molecules to produce reactive oxygen species, which selectively target microorganisms without damaging the host tissue.^10^ It has been used effectively in the past as a treatment modality for different oral diseases, including lichen planus, periodontitis, peri-implant mucositis, and fungal infections.^8,11^ Lately, it has been advocated to use PDT for treating candida-associated DS.^13,14^ The major advantage of employing PDT, unlike conventional antifungal drugs, is that retaining high drug dosage levels is not compulsory. Different studies have shown the effectiveness of PDT against recurrent infections, due to resistant *Candida* strains.^8,25,32^ A recent study by Ribeiro et al, verified that PDT could contribute to reducing the *C. albicans* count for denture disinfection as well.^32^ Moreover, Mima et al, also successfully managed 5 patients suffering from DS with PDT.^25^

Oral health-related quality of life is a measure based on patient-reported outcomes. It is used to assess an individual’s perceptions, predilections, and specific desires to promote improved clinical decision-making.^36^ Studies have shown that the patients treated with conventional complete dentures may seem to have more OHRQoL impairments than the ones who do not use any prosthesis. The continuous residual ridge resorption affects the stability and retention of the prosthesis, which in turn compromises the patient’s adaptation and comfort. On the contrary, the implant-supported prosthesis has undeniably become a much better treatment option for edentulous patients. Multiple studies have reported better OHRQoL in patients who were treated with implant-supported overdentures (IOD) rather than traditional dentures.^25^

Despite the fact that our understanding of denture stomatitis and implant overdenture prostheses has improved in recent years, it is essential to highlight the relevance of this study in the context of recent scientific evidence. Recent studies by Al-Aali et al^3^ and Abuhajar et al^1^ have highlighted the growing concern over the prevalence and impact of denture stomatitis in implant overdenture patients. These findings emphasise the urgent need to explore innovative treatment approaches, such as photodynamic therapy (PDT), that could offer more effective and patient-centered solutions. The current study may have significant implications for the management of DS and the optimisation of OHRQoL outcomes among IOD patients. It is hypothesised that OHRQoL will significantly improve after PDT treatment in patients with IOD suffering from DS. The current study aimed to determine the impact of PDT on OHRQoL among denture stomatitis patients with implant overdenture prostheses.

## Materials and Methods

This study was reviewed and approved by ethics committee of the specialist dental practice and clinical research center. Consent for voluntary participation was obtained from all the participants before enrolling them. They were allowed to leave the study without consequences at any point in time.

The patients were recruited from a specialist dental practice according to specific selection criteria. Patients 45 to 70 years old bearing an implant-retained overdenture and suffering from DS (burning sensation in the mucosa, erythema, and edema of the mucosal tissue) were included. Patients with any underlying systemic conditions, such as diabetes, heart disease, cancer, or immunological disorders were excluded from the sample set. In addition, pregnant women, patients with a history of routine illnesses, polypharmacy, and antibiotic use in the last 2 months were also excluded.

The *Candida* spp. were identified and confirmed by microbiological culturing. The swabs were attained from the surface of the maxillary IOD and palatal mucosa of the patients. They were placed in a test tube with 5 ml of 0.9% sterile saline and mixed by vortexing to extract the species. They were then incubated at 37°C for 24 h to evaluate the types and count of the species. A culture medium (CHROMagar) was used to identify the texture, colour and morphology of the species. Further, Gram staining was also carried out to identify the colonies of *Candida* spp. *Candida* counts from the palate and denture surfaces, estimated as colony-forming units (CFU/ml), were assessed at baseline, 15, 30, and 60 days.

**Fig 1:**
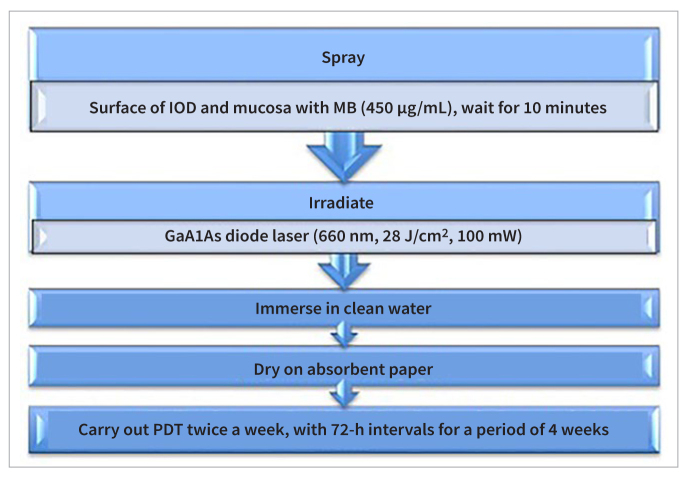
Flow diagram of the protocol for photodynamic therapy adopted.

Photodynamic therapy was carried out according to the protocol explained in Fig 1. The photodynamic therapy (PDT) treatment in this study utilised methylene blue as a photosensitiser. The protocol involved spraying the methylene-blue solution (450 μg/ml) onto the surface of the implant overdenture (IOD) and mucosa, followed by a 10-min waiting period. Laser irradiation was performed using a GaAlAs diode laser (Velas Medical Diode Laser, GIGAALASER; Wuhan, Hubei, China) with settings of 660 nm, 28 J/cm^2^, and 100 mW. Post-treatment, the area was immersed in clean water and dried with absorbent paper. PDT sessions were conducted twice a week with 72-h intervals over a 4-week period.^6^

A questionnaire was used for data collection. It included the demographic details of the patients, i.e., age, gender, education, marital, socioeconomic status, oral habits, and smoking status. In addition, the Oral Health Impact Profile-EDENT (OHIP-EDENT) scale was used to assess the OHRQoL of all patients before and after PDT application. The OHIP-EDENT is scored between 0 and 76, and the higher the score, the lower the OHRQoL. The questionnaires were available in English and Arabic languages. The domains assessed in the questionnaire were functional limitation (FL), physical pain (P1), psychological discomfort (P2), physical disability (D1), psychological disability (D2), social disability (D3), and handicap (H). The OHIP-DENT questionnaire included 19 items and 5 possible responses: never (1), hardly ever (2), occasionally (3), fairly often (4), and very often (5).

Data were tabulated and assessed using the statistical program for social sciences (SPSS Version 21, IBM; Armonk, NY, USA). Descriptive statistics and a t-test were used to assess demographic data and OHIP responses. The Shapiro-Wilk test was carried out to evaluate the normal distribution of the data. Moreover, *Candida* CFU comparison among groups was performed with the Wilcoxon test.

## Results

The participants’ ages ranged from 45 to 70 years with a mean value of 58.5 ± 10.7 years. The majority, 35 (66.0%), were males, while 18 (34.0%) were females. Of all the participants, 53 (100%) were married. Moreover, 13 (24.5%) had a primary/middle school education, 21 (39.6%) had a high school education, and 9 (17.0%) had a college education. None of the participants were illiterate, while 10 participants (18.9%) did not respond to the education question. The majority of the participants, 22 (41.5%), had a low SES, while 18 (34.0%) and 9 (17.0%) had middle and high SES, respectively. On further questioning regarding the habits, 17 (32.1%) participants reported smoking cigarettes, 15 (28.3%) reported water pipe smoking and 8 (15.1%) used electronic cigarettes. Furthermore, the majority – 30 (56.6%) participants – revealed that they currently smoked as well, whereas 10 (18.9%) were former smokers and 13 (24.5%) were non-smokers, as shown in Table 1.

**Table 1:** General characteristics of the participants

Factor	Variable	n (%)
Gender	Male	35 (66.0%)
	Female	18 (34.0%)
Marital status	Unmarried	0 (0%)
	Married	53 (100%)
Education	Illiterate	0 (0%)
	Primary/Middle	13 (24.5%)
	High school	21 (39.6%)
	College	9 (17.0%)
	No response	10 (18.9)
Socioeconomic status	Low	22 (41.5%)
	Middle	18 (34.0%)
	High	9 (17.0%)
Habits	Cigarette smoking	17 (32.1%)
	Waterpipe smoking	15 (28.3%)
	Electronic cigarette	8 (15.1%)
	None	13 (24.5%)
Smoking	Past smoker	10 (18.9%)
	Smoker	30 (56.6%)
	Nonsmoker	13 (24.5%)

**Fig 2:**
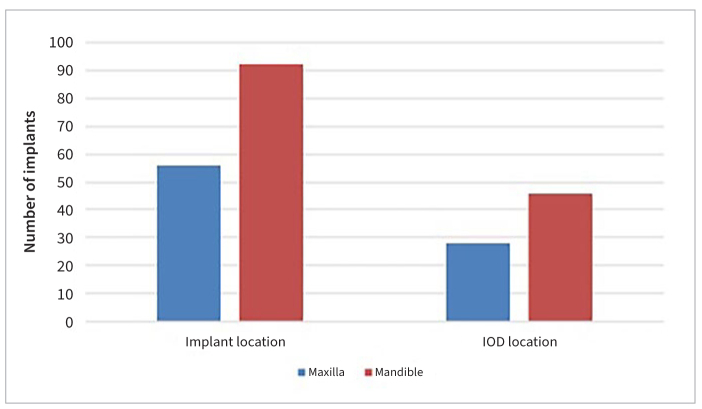
Distribution of dental implants and IODs according to location.

A total of 56 (37.8%) dental implants were used to support 28 (37.8%) IODs in the maxilla, and 92 (62.2%) implants were placed in the mandible to support 46 (62.2%) IODs, as depicted in Fig 2.

Additionally, at baseline, the overall mean *Candida* CFU/ml were quite high in the IODs samples, 37.2 ± 15.8 as compared to the palatal mucosa samples 5.1 ± 2.3. After PDT treatment, a significant reduction was noted in the mean *Candida* CFU/ml on both the mucosal surface and IOD at all follow-up visits. Moreover, a statistically significant difference in mean CFU/ml was observed on days 15, 30, and 60 in palatal mucosa samples after the PDT when compared with baseline (p < 0.05). Similarly, a statistically significant difference in mean CFU/ml was also observed on days 15, 30, and 60 in IOD samples after PDT when compared with baseline (p < 0.05). Additionally, a statistically significant difference in mean CFU/ml was also noted at day 15 in IOD samples after PDT when compared with day-30 values (p < 0.05), as presented in Table 2.

**Table 2:** Distribution of *Candida* CFU/ml found on mucosal surface and dentures after PDT application

DS Trt		Follow-up			
		Day 0	Day 15	Day 30	Day 60
PDT	Mucosal surface	5.11 ± 2.30	3.71± 2.05*	3.30± 2.10*	3.40± 2.3*
	IOD	37.17 ± 15.8	30.4±13.65*	27.81±15.61*‡	25.05± 10.48*

* Significant difference from baseline (p < 0.05). ‡ Significant difference from day 15 (p < 0.05). DS Tr: denture stomatitis treatment.

The impact of photodynamic therapy on various domains of the OHIP-EDENT showed a statistically significant improvement post-PDT treatment (Table 3). Functional limitation (FL) demonstrated a mean reduction from 13.0 ± 4.3 to 10.8 ± 3.2, indicating enhanced daily functionality. Similarly, physical pain (P1) decreased from 13.3 ± 3.4 to 10.2 ± 3.1, reflecting notable pain reduction. Psychological discomfort (P2) was also ameliorated, with a mean score reduction from 6.6 ± 1.7 to 4.4 ± 1.2. Psychological disability (D2) and social disability (D3) exhibited statistically significant reductions in scores. However, physical disability (D1) and handicap (H) did not show statistically significant changes post-PDT treatment.

**Table 3:** Comparison of pre- and post-PDT scores of OHIP-EDENT domains

Domains	Pre-PDT score Mean (SD)	Post-PDT score Mean (SD)	p-value
Functional limitation (FL)	13.0 ± 4.3	10.8 ± 3.2	< 0.05 *
Physical pain (P1)	13.3 ± 3.4	10.2 ± 3.1	< 0.05 *
Psychological discomfort (P2)	6.6 ± 1.6	4.4 ± 1.2	< 0.05 *
Physical disability (D1)	8.6 ± 3.1	7.0 ± 2.2	> 0.05
Psychological disability (D2)	5.7 ± 1.2	4.3 ± 1.8	< 0.05*
Social disability (D3)	5.2 ± 1.2	4.5 ± 1.4	< 0.05*
Handicap (H)	3.8 ± 2.3	3.4 ± 2.1	> 0.05
Total	56.2 ± 3.4	44.6 ± 2.1	< 0.05*

* Statistical significance set at p < 0.05. PDT: photodynamic therapy; SD: standard deviation.

## Discussion

Denture stomatitis is an extremely prevalent inflammatory condition observed in the palatal mucosa of denture wearers. It is strongly associated with *Candida* species and has a negative impact on the OHRQoL. If left untreated, it can promote and aggravate systemic diseases as well.^13,14^ This study found that the overall mean *Candida* CFU/ml was higher in samples taken from the surface of the IOD at baseline, 37.2 ± 15.8, as compared to the mucosal surface with 5.1 ± 2.3. Comparable results were observed in a study by Mima et al,^26^ where 97.5% and 70% of the swabs taken from the denture’s intaglio surface and the palatal mucosa revealed *Candida* growth. This could be explained by various studies which report that *Candida* species show different degrees of cell surface hydrophobicity, which increases their affinity to adhere to and colonise acrylic surfaces.^4,15,20^ Mima et al^25^ and Ribeiro et al^32^ also noted an increased *Candida* count on the denture surface than the mucosal surface. Moreover, it has also been suggested that the lower *Candida* count could be due to the sampling technique implemented. According to Dalle et al,^12^ *Candida* spp. can invade the oral epithelium over a period of 48 h through hyphal penetration into the superficial epithelium. Hence, the swab sampling technique might underestimate the real burden present on the oral mucosal surface. Therefore, other sampling techniques, including the oral rinse method using saline or sterile water, are suggested.^26^

In this study, considerable reduction in the mean *Candida* CFU/ml on both the mucosal surface and IOD reduction was observed after PDT treatment at all follow-up visits. This clearly shows the effectiveness of PDT in managing DS. Moreover, the reduction in *Candida* CFU/ml on both the mucosal surface and denture at day 15 post-treatment was particularly noteworthy, as it demonstrates that PDT can provide early therapeutic benefits to DS patients. Similar findings were reported by other authors as well.^14,29^ Moreover, other studies also reported that no statistically significant difference was found between the efficacy of PDT and conventional antifungal therapy.^2,5,12,23,24,26^ The latter has the disadvantage of developing resistant species if used frequently and for a prolonged duration.^5,12,26^ Labban et al^23^ found PDT to be effective against cigarette smokers suffering from DS as well. Afroozi et al^2^ advocate the use of a PDT-nystatin combination for effective results. Those authors found that the mean reduction achieved by the combination was statistically significantly higher than nystatin alone (p < 0.0001).^2^ Scwingel et al^34^ demonstrated the effectiveness of PDT against oral candidiasis in HIV-infected patients. They determined that although fluconazole was effective, relapse occurred shortly after the treatment was discontinued, whereas PDT eradicated 100% of the colonies, and no recurrence of candidiasis was noted up to 30 days after irradiation.^34^

Moreover, photosensitiser plays a significant role in the clinical efficacy of PDT in oral pathologies. In this study, methylene blue was used as a photosensitiser, which was effective. A few other studies also used methylene blue and obtained similar results.^5,14,34^ Afroozi et al^2^ and Labban et al^23^ achieved similar results using indocyanine green-mediated PDT and Rose Bengal and Curcumin-mediated PDT. One study showed that although photodithazine-mediated PDT resulted in clinical resolution of DS, recurrence was observed in all patients during the follow-up period.^8^

Kilic et al^21^ evaluated the prevalence of DS in IODs and found that DS developed in 100% and 71.4% of patients using bar-retained OD and locator-retained OD, respectively. DS can have a negative effect on the OHRQoL as it may present with inflammation and a burning sensation of the mucosal tissues along, with altered taste and halitosis.^18,24,27,33^ No study has been conducted yet that has evaluated the OHRQoL among DS patients with IOD treated with PDT. In our study, it was observed that all domains of OHIP-EDENT except for physical disability and handicap had statistically significantly improved in mean scores after PDT treatment; therefore, our hypothesis was accepted. The total OHIP-EDENT score after PDT showed a statistically significant improvement of 11.6, suggesting that PDT treatment can positively impact OHRQoL for patients with DS. Similar results were achieved when PDT was used to treat other oral pathologies with similar presentations. Cosgarea et al^11^ and Labban et al^22^ observed a statistically significant improvement in OHRQoL after PDT in patients suffering from a burning sensation related to oral lichen planus, caries, pericoronitis, and halitosis.^8,11,22^ As PDT reduced the *Candida* burden of mucosal tissues and IOD, thereby increasing the OHRQoL of the patients, it is therefore suggested that PDT should be used in dental clinics as an alternative to antifungal agents.

In comparison to denture stomatitis in complete-denture patients, the current study’s findings among DS patients with implant overdentures suggest that photodynamic therapy holds promise as a more effective treatment option. The study outcomes revealed statistically significant improvements in various domains of oral health-related quality of life, such as functional limitation, physical pain, and psychological discomfort, following PDT. These findings indicate that PDT may offer advantages over traditional antifungal treatments often employed for DS in complete-denture patients. However, it is crucial to emphasise the need for further research to directly compare PDT to antifungal therapies and explore its long-term sustainability and potential adverse effects. Future studies should provide valuable insights into the most suitable treatment modalities for denture stomatitis in different patients and guide clinical decision-making.

Moreover, this study highlights the significance of patient-centered care and the need to consider OHRQoL in the management of DS among IOD patients. Although the methodology of the present study was performed with great care, certain limits must be mentioned: the severity of DS was not identified, leaving unanswered the question of treatment response in cases of differing severity; the association with oral and denture hygiene as well as other risk factors, including smoking, was not examined in the current study. Future studies should take these risk factors, in addition to clinical efficacy in terms of the resolution of inflammation, into consideration. The current study only focused on PDT, wherease further studies should be conducted to evaluate the impact of other interventions on OHRQoL.

Based on the study outcome, clinical recommendations include considering photodynamic therapy as an effective treatment option for denture stomatitis in patients with implant overdentures, emphasising individualised treatment plans, educating patients on denture hygiene and maintenance, implementing regular follow-up assessments, and encouraging further research to refine protocols and explore the potential of PDT in improving the oral health-related quality of life.

## Conclusion

Photodynamic therapy (PDT) has a statistially significantly positive impact on the oral health-related quality of life (OHRQoL) among denture stomatitis patients with implant overdenture prostheses. Moreover, PDT can be used as an effective treatment option for the management of denture stomatitis in patients with implant overdenture prostheses.
